# Successful use of extracorporeal membrane oxygenation in the reversal of cardiorespiratory failure induced by atonic uterine bleeding: a case report

**DOI:** 10.1186/1752-1947-8-23

**Published:** 2014-01-27

**Authors:** Taiga Itagaki, Mutsuo Onodera, Nao Okuda, Emiko Nakataki, Hideaki Imanaka, Masaji Nishimura

**Affiliations:** 1 Department of Emergency and Critical Care Medicine, Tokushima University Hospital, 3-18-15 Kuramoto-cho, Tokushima 7708503, Japan; 2 Department of Emergency and Disaster Medicine, Tokushima University Hospital, 3-18-15 Kuramoto-cho, Tokushima 7708503, Japan

**Keywords:** Atonic uterine bleeding, Cardiorespiratory failure, Extracorporeal membrane oxygenation

## Abstract

**Introduction:**

Although extracorporeal membrane oxygenation has made sufficient progress to be considered for the management of life-threatening cardiac and respiratory failure, the risk of hemorrhagic complications may outweigh the benefits for patients with bleeding tendencies. We report, to the best of our knowledge, the first case of successful treatment by extracorporeal membrane oxygenation, without any hemorrhagic complications, of postpartum cardiorespiratory failure after massive uterine bleeding.

**Case presentation:**

A 25-year-old Japanese woman experienced massive atonic bleeding after delivering her second baby. Recovery from hemorrhagic shock was managed by conservative treatments, but she developed decompensated heart failure and refractory hypoxia. Because we could not obtain hemodynamic stability and proper oxygenation even with high doses of catecholamines and maximal ventilator settings, we administered venoarterial extracorporeal membrane oxygenation, whereupon her hemodynamic status immediately stabilized. After 72 hours of support without major bleeding, extracorporeal membrane oxygenation was successfully withdrawn.

**Conclusion:**

Even in cases of obstetric bleeding, if clotting status is stringently monitored, extracorporeal membrane oxygenation can be considered as an ultimate means of life support.

## Introduction

Extracorporeal membrane oxygenation (ECMO) has rapidly developed and is widely used both for circulatory support and for the treatment of acute respiratory distress [1,2]. Although ECMO is a candidate therapy for life-threatening cardiorespiratory failure, there are few reports of using ECMO in obstetric cases with massive bleeding. The reluctance to use ECMO likely stems from the necessity of taking anticoagulants, which is likely to aggravate bleeding. We present the case of a patient with postpartum cardiorespiratory failure after severe atonic uterine bleeding, who was successfully treated using ECMO without any hemorrhagic complications.

## Case presentation

A 25-year-old Japanese woman (155cm, 52kg) delivered her second baby (41 weeks and 0 day’s gestation). She did not have any appreciable pre-existing diseases including cardiac morbidities. After delivery, she showed poor uterine contraction and, despite continuous administration of oxytocic agents, massive bleeding. Her total blood loss was estimated to be about 5000mL. On admission, after transfer to our hospital, her blood pressure was 80/40mmHg and heart rate was 170 beats per minute. Laboratory data revealed severe disseminated intravascular coagulation (DIC), with a platelet count of 13×10^3^/mm^3^, 64μg/dL of fibrin/fibrinogen degradation products, and unmeasurable international normalized ratio of prothrombin time and fibrinogen. After her trachea was intubated, she developed ventricular fibrillation due to persistent hypotension. Spontaneous circulation was restored by a single defibrillating shock.

Despite local and systemic administration of oxytocic agents and continuous bimanual compression of her uterus, atonic bleeding persisted. Her uterine contraction improved gradually and the bleeding was under control eight hours after admission. She recovered from the DIC (platelet count, 114×10^3^/mm^3^; fibrin/fibrinogen degradation products, 22μg/dL; international normalized ratio of prothrombin time, 1.06; and fibrinogen, 220mg/dL). Her blood loss for the first day at our hospital was estimated to be 18,000mL and she received 40 units of red-blood-cell concentrates (140mL/unit), 60 units of fresh frozen plasma (80mL/unit) and 40 units of platelet concentrates (20mL/unit). Altogether, she received 40 units of oxytocin and 0.4mg of methylergometrine.

**Figure 1:**
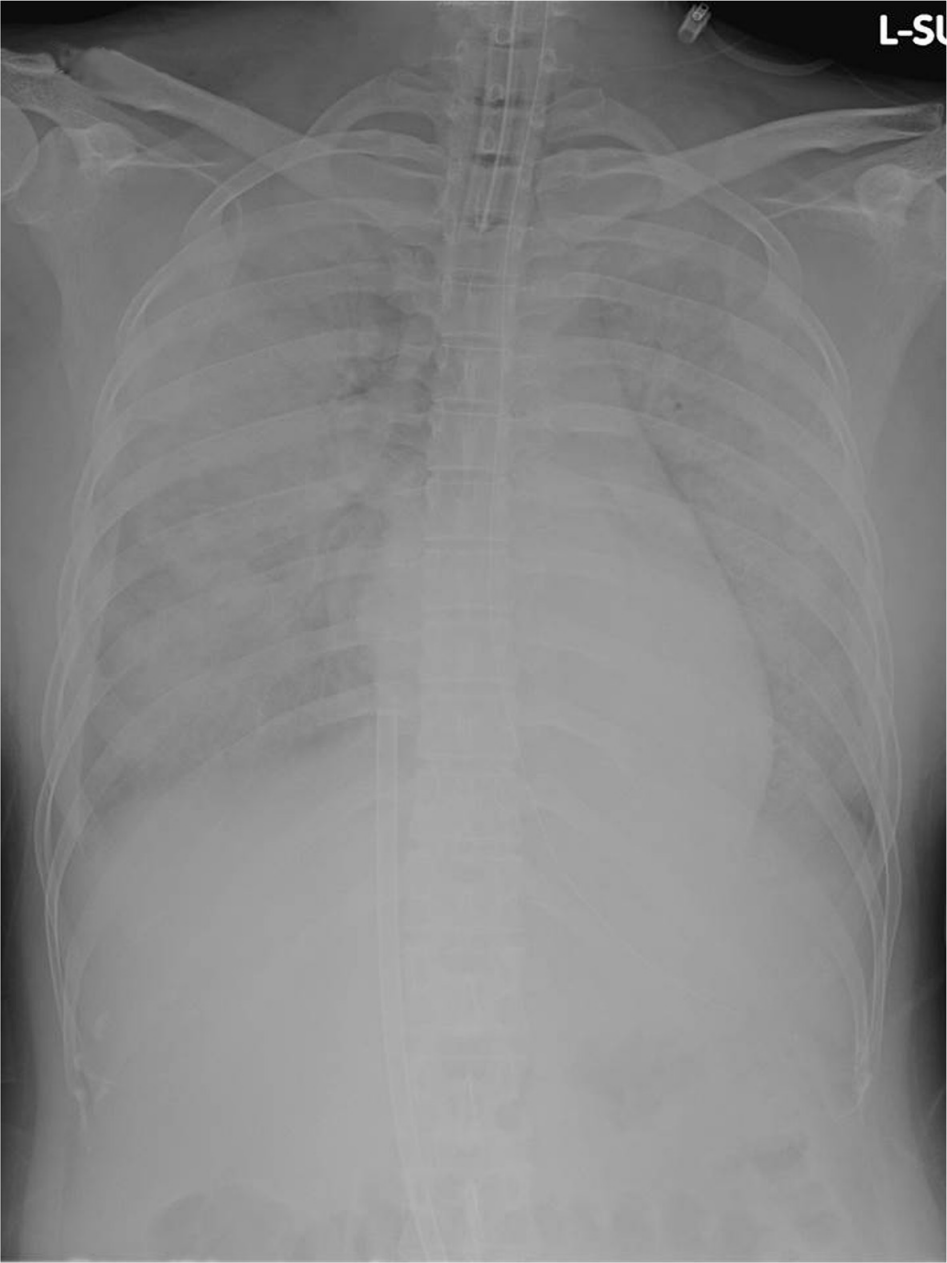
**Chest X-ray at the beginning of extracorporeal membrane oxygenation.** Chest X-ray shows diffuse white-out of the lungs. A 19-Fr drainage cannula is seen inserted into the inferior vena cava.

On the second day of her admission, her systolic blood pressure remained at around 80mmHg, and her heart rate was over 140 beats per minute. An echocardiography showed severe diffuse hypokinesis of her left ventricle with epinephrine (0.05μg/kg/min) and norepinephrine (0.1μg/kg/min). Moreover, she developed severe hypoxia, her arterial oxygen tension (PaO_2_) to fraction of inspired oxygen (F_I_O_2_) ratio (P/F ratio) was 93.9 at F_I_O_2_ 1.0 and positive end-expiratory pressure was 14cmH_2_O. Her oxygenation deteriorated further to P/F ratio 53.8 (F_I_O_2_ 1.0, positive end-expiratory pressure 14cmH_2_O). It was life-threatening and, consequently, we decided to initiate cardiopulmonary support by venoarterial ECMO (Figure 1). Her left femoral vein and artery were surgically cannulated with 19-Fr drainage and 15-Fr return cannulae, respectively. Table 1 shows the clinical course of the ECMO. We used an ECMO circuit with non-heparin coating (biocompatible coating). Rotational frequency of the centrifugal pump was controlled to ensure an ECMO flow of 3L/min. Infusion of unfractionated heparin was initiated at a rate of 400 units per hour and regulated to obtain 160 seconds of activated clotting time (ACT) and not to exceed 180 seconds. Her ACT was measured every four hours.

**Table 1:** Clinical course for the extracorporeal membrane oxygenation

		**One hour before ECMO**	**ECMO time (hour)**							**One hour after ECMO**
			**0**	**12**	**24**	**36**	**48**	**60**	**72**	
Vital signs	Mean blood pressure (mmHg)	63	86	80	104	94	99	80	92	87
	Heart rate (/min)	170	110	115	106	107	107	125	109	110
	SpO_2_, R/L (%)	-/85	100/100	100/100	96/97	98/100	97/98	99/100	96/97	100/100
Mechanical ventilation	Mode	A/C (PC)	⇒	⇒	⇒	⇒	⇒	⇒	⇒	⇒
	F_I_O_2_	1.0	1.0	0.6	⇒	⇒	0.4	⇒	⇒	1.0
	PC/positive end-expiratory pressure (cmH_2_O)	18/14	12/12	12/10	12/8	⇒	⇒	⇒	⇒	16/8
ECMO	Sweep gas (L/min)		3	2	⇒	⇒	⇒	1.8	1.5	
	F_I_O_2_		1.0	0.8	⇒	⇒	⇒	⇒	⇒	
	Pump rotational speed (round/min)		2500	2700	⇒	2400	⇒	⇒	1900	
	Pump flow (l/min)		3.0	3.4	⇒	2.8	2.7	2.5	1.3	
	Serum leakage		-	-	-	-	±	+	+	
	Uterine bleeding		-	-	-	-	-	-	-	
	Remarks					SvO_2_; 82%		Metallic sound + (pump)		
	Unfractionated heparin (units/h)	0	400	600	⇒	⇒	⇒	1000	⇒	0
	Activated clotting time (s)		257	170	179	183	181	162	165	
Arterial blood gas analysis	pH	7.39	7.5	7.45	7.4	7.44	7.41	7.46	7.48	7.57
	PaO_2_ (mmHg)	53.8	410.3	118.5	66.1	169.5	76	118.6	131.8	487.1
	PaCO_2_ (mmHg)	51.8	33.4	43.9	48.2	40.2	45.4	42.3	39	29.5
Coagulation	Platelets (×10^3^/mm^3^)	109		61		39		50		89
	International normalized ratio of prothrombin time	0.98		1.09		0.94		0.91		0.93
	Activated partial thromboplastin time (s)	31.4		53.6		68.1		50.6		34.9
	Fibrinogen (mg/dL)	278		438		619		672		504
	Anti-thrombin III activity (%)	92.9		63.1		53.2		77.4		67.3

A/C, assist and control; ECMO, extracorporeal membrane oxygenation; F_I_O_2_, fraction of inspired oxygen; L, left finger; PC, pressure control; R, right finger; SvO_2_, oxygen saturation of venous drainage blood.

After initiation of ECMO, our patient’s SpO_2_ reached 100% and her heart rate decreased from 160 to 110 beats per minute. The infusion of catecholamines was discontinued and the mechanical ventilatory support was gradually decreased. Furosemide was administered and a negative fluid balance of −2900mL was achieved during the 48 hours after ECMO. After 60 hours of support, the rotational frequency of the centrifugal pump was decreased by 30 percent. With stable hemodynamics, our patient was successfully weaned from ECMO after 72 hours of support. Four units of red-blood-cell concentrates, 10 units of platelet products and 1500 units of human anti-thrombin III were transfused and no major bleeding was detected during ECMO. Tracheal extubation was performed 42 hours after withdrawal of the ECMO (day 7). Our patient left our intensive care unit on day 10.

## Discussion

Using venoarterial ECMO, we successfully treated a patient with cardiorespiratory failure due to atonic uterine bleeding. While the literature contains examples of ECMO support being provided for peripartum women with conditions such as acute respiratory distress syndrome caused by H1N1 influenza [3,4], peripartum cardiomyopathy [5], amniotic fluid embolism [6] and transfusion-related acute lung injury [7], we found little mention of ECMO being used to manage peripartum patients with massive bleeding [8]. Generally, use of ECMO is indicated when there is life-threatening cardiac and respiratory failure and no other treatment is likely to be successful. It is used as temporary support, usually while awaiting recovery of organ functions [1]. Even with high doses of catecholamines and mechanical ventilation, our patient was unable to maintain cardiac contractility and oxygenation.

Because she showed no evidence of any pre-existing cardiac morbidities, we considered peripartum cardiomyopathy, amniotic fluid embolism, acute myocardial damage due to persistent hypotension and ventricular fibrillation, and adverse effects of oxytocic agents or catecholamines to be the cause of her decompensated heart failure. Since peripartum cardiac dysfunction is usually reversible, survival being secured by forms of mechanical cardiorespiratory support such as use of an intra-aortic balloon pump, a left ventricular assist device or ECMO [9], we considered the feasibility of ECMO. After ECMO, her cardiovascular status recovered quicker than we expected, and we supposed transient myocardial stunning or amniotic fluid embolism to be the possible causes of her hypoxemia.

The most common complication of ECMO is bleeding [1,2]. For this reason, application of ECMO to patients with hemorrhagic tendencies requires careful consideration. In our patient, controlled bleeding and her DIC profiles at the initiation of ECMO were key to the successful ECMO. Reyftmann and colleagues reported the case of a patient with refractory cardiac decompensation due to atonic bleeding after Cesarean delivery. Their patient required another operation and a massive transfusion to control intra-abdominal bleeding during ECMO [8]. Even so, the only bleeding stated as a contraindication in the Extracorporeal Life Support Organization Guidelines is intracranial bleeding, and physicians are urged to be flexible in their response [10]. The guidelines recommend ECMO when ACT values are up to 1.5-fold of normal, platelet count can be maintained at greater than 80×10^3^/mm^3^, and fibrinogen is kept within the normal range (250 to 300mg/dL). It also mentions that circuits and oxygenators with heparin surface coatings may be helpful in the management of patients with bleeding. In cases of massive bleeding, it is essential to avoid unnecessary surgical procedures and to make a maximum effort to stop the bleeding. Recently, Lamb and colleagues reported on five patients undergoing ECMO with massive bleeding managed successfully with an anticoagulation protocol without thrombotic complications [11]. In our case, although our patient’s bleeding was controlled, we tightly regulated the ACT range between 160 and 180 seconds owing to concerns about rebleeding from her uterus. We managed to complete the treatment without any major bleeding.

Blood clots are another frequently encountered circuit-related complication of ECMO [1,2,12]. With our patient, a metallic sound was heard from the centrifugal pump during the final few hours of ECMO and blood clots were assumed to have been generated. This reminded us of the importance of tight anticoagulation control and careful examination of the entire extracorporeal circuit, especially where non-heparin coatings are used or when the anticoagulation target is reduced for patients with bleeding tendencies.

## Conclusions

After prompt initiation of ECMO, we managed, without any hemorrhagic complications, to reverse cardiorespiratory failure caused by atonic uterine bleeding. As long as meticulous examination of clotting status is carried out before and during the procedure, ECMO should be considered as an ultimate means of life support, even in cases of obstetric bleeding.

## Consent

Written informed consent was obtained from the patient for publication of this case report and any accompanying images. A copy of the written consent is available for the review by the Editor-in-Chief of this journal.

## Abbreviations

ACT: activated clotting time; DIC: disseminated intravascular coagulation; ECMO: extracorporeal membrane oxygenation; FIO2: fraction of inspired oxygen; PaO2: arterial oxygen tension; P/F ratio: ratio of arterial oxygen tension to fraction of inspired oxygen.

## Competing interests

The authors declare that they have no competing interests.

## Authors’ contributions

TI was a major contributor in writing the case report. TI, MO, NO and EN were equally responsible for data collection. HI and MN provided critical revision of the case report. All authors read and approved the final case report.
